# Molecularly-guided spatial proteomics captures single-cell identity and heterogeneity of the nervous system

**DOI:** 10.1101/2025.02.10.637505

**Published:** 2025-02-10

**Authors:** Sayan Dutta, Marion Pang, Gerard M. Coughlin, Sirisha Gudavalli, Michael L. Roukes, Tsui-Fen Chou, Viviana Gradinaru

**Affiliations:** 1 Division of Biology and Biological Engineering, California Institute of Technology, Pasadena, CA 91125; 2 Aligning Science Across Parkinson’s (ASAP) Collaborative Research Network, Chevy Chase, MD 20815; 3 Division of Physics, Mathematics, and Astronomy, California Institute of Technology, Pasadena, CA 91125; 4 Division of Engineering and Applied Science, California Institute of Technology, Pasadena, CA 91125

## Abstract

Single-cell proteomics is an emerging field with significant potential to characterize heterogeneity within biological tissues. It offers complementary insights to single-cell transcriptomics by revealing unbiased proteomic changes downstream of the transcriptome. Recent advancements have focused on enhancing proteome coverage and depth, mostly in cultured cell lines, and a few recent studies have explored the potential of analyzing tissue micro-samples but were limited to homogenous peripheral tissues. In this current work, we utilize the power of spatial single cell-proteomics through immunostaining-guided laser capture microdissection (LCM) coupled with LC-MS to investigate the heterogenous central nervous system. We used this method to compare neuronal populations from cortex and substantia nigra, two brain regions associated with motor and cognitive function and various neurological disorders. Moreover, we used the technique to understand the neuroimmune changes associated with stab wound injury. Finally, we focus our application on the peripheral nervous system, where we compare the proteome of the myenteric plexus cell ganglion to the nerve bundle. This study demonstrates the utility of spatial single-cell proteomics in neuroscience research toward understanding fundamental biology and the molecular drivers of neurological conditions.

## Introduction

The nervous system comprises one of the most complex tissues in the human body(1). Functionally and anatomically segregated neuronal subpopulations and mosaic coexistence of glial cells (i.e., astrocytes, microglia, and oligodendrocytes) contribute to the intrinsic heterogeneity of the nervous tissue. Therefore, preserving spatial context is crucial in tissue studies, particularly where gradients arise from metabolic processes(2, 3) or during neurological disorders such as neurodegenerative diseases (ND) (4). For instance, at the later stages of Parkinson’s disease (PD), the 2^nd^ most common ND, only 1–2% of the sparsely situated neurons in the cortex show the presence of pathological Lewy body (LB) aggregates(5). Identifying the perturbations in these specific neurons that bulk omics approaches fail to capture is essential for advancing our understanding of the disease.

Numerous spatial analysis techniques have been employed to study cell type specificity and neurodegenerative diseases in a spatially resolved context, though most focus on transcriptomic insights(4, 6). While protein-focused methods, such as imaging mass cytometry (IMC)(7), cyclic immunofluorescence (cycIF)(8) and co-detection by indexing (CODEX)(9) provide meaningful insight, they are typically restricted to a predefined set of targeted proteins, limiting their scope in capturing the complexity of the complete proteomic landscape. Other studies(10, 11) have employed spatial mass spectrometry methods for unbiased proteomic analysis, such as extracting 1 mm tissue cores from distinct brain regions(12) or digesting 5 mm droplets directly on tissue slides. While these approaches provide a comprehensive view of the proteome, their sampling areas are disproportionately large relative to the spatial scale of brain cells, resulting in analysis comprising heterogeneous mixtures of cell types and tissue components, including multiple vessels, extracellular space, and cellular interfaces – thus limiting resolution and specificity. This limitation precludes proteomic analysis of molecularly and/or spatially defined cell types, especially rare ones.

Recent advances in mass-spectrometry-based spatial proteomics of low-input tissue samples, especially at the single-cell level, offer new potential for exploring spatial heterogeneity and proteomic diversity. Advancements in single-cell proteomics studies have primarily focused on cultured cell systems, through improvements in instrumentation and sample preparation(13, 14, 15, 16, 17, 18), with only a few recent studies exploring the potential of analyzing tissue micro-samples. Scalable frameworks combining high-content imaging, sample isolation via laser-capture microdissection (LCM) or laser ablation, and ultrasensitive MS approaches have been developed for ultra-low-input archival tissue samples by various groups(2, 19, 20). These advancements allow for detailed spatial proteome profiling across contexts such as liver zonation or spatially resolved proteomes in tonsil tissue, which reveal sites of clonal B cell expansion and somatic hypermutation(2, 19).

This study pioneers the integration of laser capture microdissection with cutting-edge single-cell proteomics (LCM-ScP) to study the complex and heterogeneous mouse nervous system while enabling the preservation of spatial biological context within. We explore the potential and constraints of this innovative approach by targeting challenging problems, including cell type heterogeneity, cell type-specific responses to injury, and proteomic differences between neuronal compartments and corroborating findings with established biological frameworks.

## Results

### Marker-guided proteomics of central nervous system (CNS) neurons yields consistent and high protein coverage across single-cell samples

**Figure 1:**
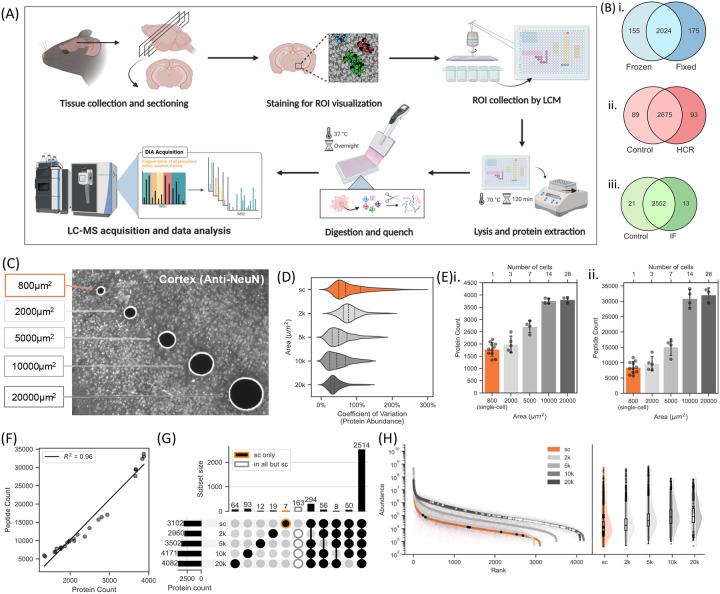
Design and optimization of molecularly-guided spatial single-cell proteomics for CNS tissue. (A) Methodology overview: Mouse brain tissue was collected, fixed, and cryo-sectioned, followed by IF staining for specific markers. Regions of interest (ROIs) were collected via LCM, followed by lysis and protein extraction into 384-well plates. Proteins were digested overnight and analyzed using LC-MS in DIA mode. (B) Protein overlaps across three conditions: (i) fixed tissue (vs. frozen), (ii) marker protein detection via IF (iii) marker mRNA detection via HCR. (C) Microscope image illustrating different areas (800 μm^2^, 2000 μm^2^, 5000 μm^2^, 10000 μm^2^, 20000 μm^2^) isolated from NeuN immunostained mice brain cortical section. (D) Coefficient of Variation (CV) of protein abundance for different areas, showing higher CVs in single cells, indicating greater heterogeneity compared to larger areas. (E) Number of high-confidence (i) proteins (q<0.01) and (ii) peptides (q<0.01) identified in samples of different areas. (F) Pearson correlations of protein identifications to peptide identifications showing linear trend (R^2^=0.97). (G) Upset plot of protein intersections between areas, with 7 proteins unique to single-cell samples and 163 proteins identified in all but single-cell samples. (H) (I) Abundance-rank quant plot showing the distribution of proteins across the dynamic range, with single-cell-specific proteins in black and all-but-single-cell proteins in white.

For unbiased mapping of proteome from single cells of fixed brain tissue, we adapted(2, 20) a workflow that integrates an immunostaining-guided selection of micro-tissue samples for downstream proteomic analysis (Figure 1A). Our workflow begins with tissue fixation, O.C.T. embedding, and cryo sectioning, followed by immunofluorescent or HCR staining to label cells of interest, facilitating pathology-guided selection for proteomic analysis. Targeted cells are then isolated with precision using LCM, enabling accurate excision of specific regions from tissue sections. The isolated cells are collected in a lysis buffer and subjected to digestion with a trypsin/Lys-C mix, a protocol optimized to enhance protein digestion efficiency, particularly in low-analyte samples(21, 22).

Before employing stain-guided isolation and processing of samples from fixed tissue, we first assessed whether tissue staining protocols impact downstream proteome coverage or introduce bias in detected peptides. Since tissue fixation is a prerequisite for staining, we evaluated whether the fixation process itself influences proteomic analysis. This evaluation, performed using data-dependent acquisition (DDA) mode on the mass spectrometer with a shotgun 1-hour gradient for rapid assessment, included comparisons between (1) fresh-frozen versus fixed tissue (2) tissue stained for protein marker using immunohistochemistry (IHC) versus unstained control, and (3) tissue stained for mRNA marker using hybridization chain reaction (HCR). Comparing frozen and fixed tissues, we observed relatively consistent proteome coverage, with 2,024 shared proteins, 175 unique to fixed tissue, and 155 unique to frozen tissue (Fig. 1B, i). However, statistical analysis revealed significant differences, and the log2 protein abundance correlation between frozen and fixed tissues was moderate (R2 = 0.70; Fig. S1A). Anti-NeuN staining on cortical tissue and HCR probes against Inositol 1,4,5-Trisphosphate Receptor Type 1 (Itpr1) mRNA on cerebellar tissue were used as proxy markers for IF and HCR staining methods, respectively. Both HCR and immunofluorescence showed substantial overlap with their respective unstained controls (Fig, 1B, ii–iii). Moreover, protein counts remained similar between stained and control samples for both staining protocols (Fig. S1B, S1C, p < 0.05). log2 protein abundance values between stained and PBS-treated samples demonstrated a strong linear correlation (R2 = 0.97 for IF and R2 = 0.95 for HCR). Panther overrepresentation analysis for GO enrichment across biological processes, molecular function, and cellular components revealed no significant enrichments, indicating that immunofluorescence staining did not introduce a detectable bias in proteome coverage (Fig. S1A-vi). Furthermore, when plotting the proteomic data on a rank-quant plot, proteins unique to stain or unique to control were biased towards the lower abundance range, a characteristic typically attributed to stochastic sampling in DDA methods (Fig S1B–C, v). This suggests that these proteins are likely the result of stochastic variability inherent to DDA rather than biases introduced by the staining protocol.

Having established that proteome analysis via LC-MS is unaffected by staining protocols, we next evaluated the impact of sample size, measured as the number of neuronal cells, using the cortex as a baseline. To facilitate this, immunofluorescence staining was used to label NeuN, enabling the identification and isolation of cortical neurons for proteomic analysis. Building on this, we aimed to test the limits of the LCM-ScP workflow for ultra-low input samples. Using LCM, we isolated tissue section areas corresponding to varying neuronal cell-body counts: single cells (~800 μm2), ~3 cells (2,000 μm2), ~7 cells (5,000 μm2), ~14 cells (10,000 μm2), and ~28 cells (20,000 μm2) (Fig. 1C, Sup. Fig. S2). It should be noted that the laser of the LCM cuts via cold ablation, to achieve a fine cut without melting tissue. Thus, the actual excised sample sizes are slightly smaller than the defined areas due to the laser’s resolution, which does not achieve single-pixel precision. Samples were analyzed using a previously optimized 1-hour LC-MS gradient with data-independent acquisition (DIA)(21, 22), as shown in Fig. 1D, E. Notably, protein identification plateaued beyond the 10,000 μm2 sample size, as the gradient was optimized specifically for single-cell applications. Our approach yielded consistent and high protein coverage across single-cell samples (n=11), with a maximum depth of 2,200 proteins quantified from a single cell. Greater heterogeneity was observed in single-cell samples, with higher coefficients of variation (CVs) compared to larger sample areas from the homogenous cortex region, highlighting the method’s utility for studying cellular heterogeneity (Fig. 1D).

Analysis of proteins across different sample areas revealed only 7 proteins unique to single-cell samples and 163 proteins consistently detected in all but the single-cell samples (Fig. 1H). Abundance-rank quant plots show the distribution of these proteins, with single-cell-specific proteins spanning a wide dynamic range, indicating that single-cell samples can detect a broad range of proteins without abundance bias. In contrast, proteins missing from single-cell samples (shown in white) were primarily low-abundance proteins, consistent with the expectation that these proteins fall below detection thresholds in single-cell samples due to MS sensitivity limitations. While DIA mitigates stochastic sampling issues, resolving single-cell DIA presents unique challenges due to a lower signal-to-noise ratio. These results establish LCM-ScP as a tool for unbiased proteomic characterization of single neurons, which we next apply to neuroscience-focused samples to demonstrate its utility.

### Single-cell proteomics can distinguish CNS neurons from different brain regions and efficiently identify region-specific neuronal markers

**Figure 2:**
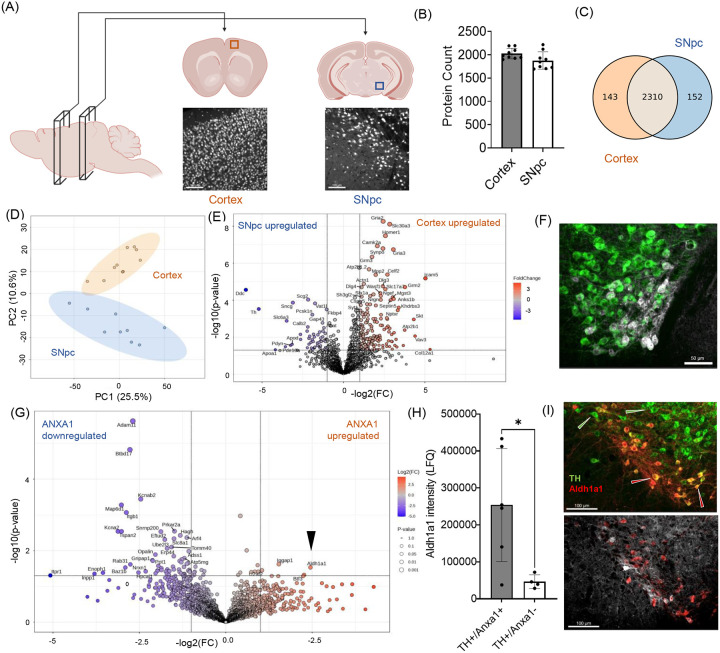
Characterization of CNS neuronal subpopulations using LCM-ScP (A) Schematic representation of the cortex and SNpc brain regions selected for comparison using LCM-ScP. Confocal microscopy images showing immunostaining of neuronal cell bodies in the cortex and SNpc. Neurons are labeled with anti-NeuN. Scale bar: 100 μm. (B) Protein counts from isolated single-cell samples obtained from cortical and SNpc regions using LCM-ScP. (n = 9 cells/group). (C) Overlap of identified proteins between cortical and SNpc samples and proteins uniquely identified in each cell type. (D) An unsupervised PCA plot of protein intensities from two sample groups showed clear clustering of the single-cell samples from the cortex and SNpc. (E) Distribution of differentially expressed proteins between the cortex and SNpc. Proteins significantly upregulated in each neuron type are highlighted, revealing region-specific protein markers. (F) Immunofluorescent staining of mice SNpc neurons using tyrosine hydroxylase (TH) as a pan-dopaminergic neuronal marker and annexin A1 (Anxa1) as a marker for the ventral subpopulation of SNpc (representative TH+/Anxa1+ neurons are indicated by arrowheads). Scale bar: 50 μm. (G) Distribution of differentially expressed proteins between the single dopaminergic neurons (TH+) isolated based on Anxa counterstain (i.e., Anxa1-positive (Anxa1+) and Anxa1-negative (Anxa1−)). (H) Mass spectrometry protein intensities of Aldehyde Dehydrogenase 1 Family Member A1 (Aldh1a1), identified as differentially regulated between the two populations (indicated by the arrowhead in Fig. G). Statistical significance was assessed using Student’s *t*-test (*p* < 0.05 is denoted by *). (I) Immunofluorescent validation of Aldh1a1 expression in midbrain dopaminergic neurons, revealing a greater proportion of Aldh1a1-positive neurons in the ventral midbrain (representative cell populations indicated by arrowheads). Scale bar: 100 μm.

We applied LCM-ScP to characterize neuronal subtypes from distinct brain regions (Fig. 2A). For this, we immuno-stained 35 μm thick PFA-fixed coronal brain slices from adult mice with pan-neuronal anti-NeuN to visualize neurons. Single neuronal cell bodies were micro-dissected from the cortex (approximately from layers IV) and the substantia nigra pars compacta (SNpc, validated with anti-tyrosine hydroxylase (TH) counterstain) for subsequent proteomic profiling.

Proteomic analysis led to the identification of approximately 2000 proteins on average per cell (Fig 2B). We identified 2310 protein groups across both neuronal populations, with an additional 143 proteins exclusively detected in cortical neurons and 152 in SNpc neurons (Fig. 2C). Unsupervised principal component analysis (PCA) revealed two distinct clusters, highlighting significant molecular differences between cortical and SNpc neurons (Fig 2D. Among the detected proteins, 345 showed statistically significant differences (p < 0.05), with 143 proteins exhibiting more than two-fold upregulation in cortical neurons and 77 proteins in SNpc neurons. This analysis identified several regionally selective proteins (Fig 2E). In cortical neurons, Calcium/Calmodulin-Dependent Protein Kinase II Alpha (Camk2a), Glutamate Ionotropic Receptor AMPA Type Subunit 2 (Gria2), Intercellular Adhesion Molecule 5 (Icam5), and Homer1 protein Homologs were enriched. In contrast, TH, Dopa Decarboxylase (Ddc), Gamma-Synuclein (Sncg), and Calbindin 2 (Calb2) were highly expressed in SNpc. To validate these proteomic findings, we cross-referenced the identified proteins with in-situ hybridization (ISH) gene expression data from the Allen Brain Atlas and performed immunohistochemistry (IHC) (Sup. Fig. S3). Additionally, we plotted the mass spectrometry-derived intensity values to showcase the heterogeneity in protein expression at the single-cell level.

Comparing proteomes between brain regions is valuable, but we also wanted to see if we could compare between molecularly defined neuronal subpopulations that are closer in terms of their function and anatomy. cell types in same region. We applied LCM-ScP to investigate. We isolated Annexin1 (Anxa1) positive TH neurons from the ventral SNpc, a neuronal population vulnerable to PD(23), and compared them to the dorsal TH+/Anxa-cells (Fig 2F). We identified many proteins selectively present in one of two populations with statistically significant differences (Fig 2G). Aldehyde Dehydrogenase 1 Family Member A1 (Aldh1a1) was identified as one of the hits upregulated in Anxa1+ neurons (Fig. 2G). This finding was confirmed by IHC (Fig. 2H) and is also supported by previous literature(23).

These findings establish LCM-ScP as a powerful approach to investigate neuronal subpopulations, offering complementary data to transcriptomics for different disorders where cell type specificity is important.

### Single-cell proteomics of non-neuronal cells in CNS can capture markers of neuroinflammation

**Figure 3.**
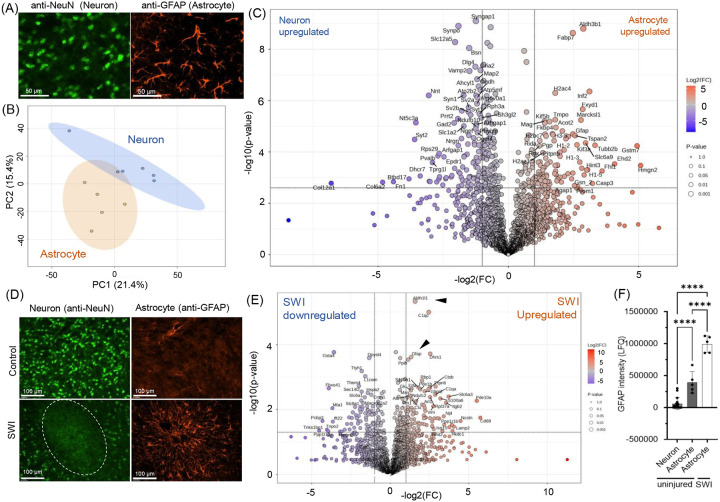
Single-cell proteomic investigation of CNS astrocytes in control tissue and during acute inflammation. (A) Representative immunofluorescent images of neuronal cells stained with anti-NeuN and astrocytes stained with anti-GFAP. Scale bar: 50 μm. (B) Unsupervised PCA plot of protein intensities showing clear clustering of single-cell proteomics samples from neuronal and astrocytic populations in the mouse brain cortex. (C) Differentially expressed proteins between neuronal and astrocytic single-cell samples. (D) Representative IHC images comparing neurons and astrocytes in uninjured brain regions versus the injury site (n=4) in an acute stab wound injury (SWI) model in mice. NeuN staining demonstrates a loss of neurons at the injury center along the needle path, while GFAP staining highlights the presence of reactive astrocytes at the injury site. (E) Differentially expressed proteins in single astrocytes isolated from uninjured brain regions versus those at the injury site. Well-validated marker proteins significantly upregulated in astrocytes from the injured tissue are annotated with black arrowheads. (F) Mass spectrometry-derived protein intensities for the reactive astrocyte marker GFAP in neurons and astrocytes, comparing samples from the injured and uninjured brain regions. Statistical significance was assessed using one-way ANOVA with Tukey’s post hoc comparison (**** indicates p-value <0.0001),

Glial cells are essential for maintaining central nervous system (CNS) homeostasis, providing structural support, and regulating neuronal function(24). They also contribute to neuroplasticity, metabolic support, and the modulation of inflammatory responses, making them central to both normal CNS function and pathological processes(25). To investigate the potential of LCM-ScP in studying non-neuronal CNS cells, we employed a directed approach to isolate and analyze glial cells, especially astrocytes. Brain sections were stained using astrocytic marker anti-glial fibrillary acidic protein (GFAP), alongside anti-NeuN to identify and isolate non-overlapping populations of astrocytes specifically (Fig. 3A). PCA plots of the proteomic data showed independent clusters with minute overlap, likely pointing to the need to increase population size (Fig. 3B). Analysis identified multiple cell type-specific proteins (Fig. 3C). Neuron-specific proteins included markers such as Bassoon (Bsn), Parvalbumin (Pvalb), Synapsin-1 (Syn1), and Discs Large MAGUK Scaffold Protein 4 (Dlg4), while astrocyte-specific proteins included Fatty Acid Binding Protein 7 (Fabp7) and GFAP.

Next, we used LCM-ScP to study neuroinflammation induced by acute brain injury. We employed a stab wound injury (SWI), a model used to study the effects of traumatic brain injury(26, 27), which causes rapid neuronal death along the needle track and the formation of a glial scar in the surrounding region (Fig. 3D). GFAP+ glial cells were isolated from around the injury site and compared with GFAP+ cells from the contralateral, non-injured hemisphere. Proteomic analysis revealed several differentially expressed proteins (Fig. 3E), including Aldehyde Dehydrogenase 1 Family Member L1 (Aldh1l1) and GFAP, both markers of astrocytic activation. Increased GFAP expression was evident in both single-cell proteomic intensity plots (Fig. 3F) and validated by IHC in multiple animals (Fig. 3D, n=4 animals).

We aimed to apply this method to study microglia in control brain tissue, using Ionized Calcium-Binding Adaptor Molecule 1 (Iba1) as a marker (Sup. Fig. S4A). However, comparisons between microglia and neurons revealed no significant differences in protein profiles and showed high overlap in PCA clusters. This overlap likely resulted from the small size of microglia (Sup. Fig. S4B), and their phagocytic activity, which may have led to the unintentional inclusion of adjacent neuronal proteins during sample collection, causing proteomic contamination. However, microglial inflammatory proteins can be detected via proteomics. Acute brain injury also initiates strong microglial ramification (Sup. Fig. S4A) alongside astrocytic activation. Therefore, single-cell astrocyte samples also consisted of upregulation of proteins associated with microglial activation, such as CD68 and CD11b (Sup Fig. S4C, (i)–(ii)) due to some proteomic contamination and irregular shapes of glia.

Together, these findings demonstrate the power of LCM-ScP for studying neuroinflammation, particularly in region-specific gliosis, a critical feature in the progression of neurological disorders such as stroke and neurodegenerative disease.

### Spatial proteomics for studying myenteric plexus neurons of the peripheral nervous system

**Figure 4:**
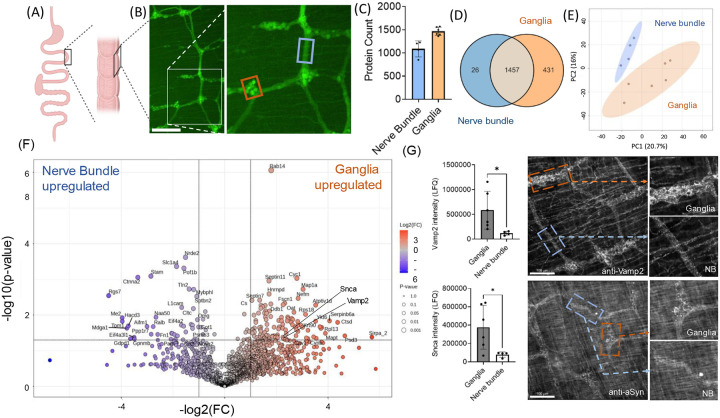
Proteomic investigation of MP ganglionic cell bodies and axon bundles of gut PNS using spatial proteomic workflow. (A) Schematic of the LMMP layer isolation, highlighting the extraction region. (B) IHC visualization of the myenteric plexus network, with neuronal staining using PGP9.5. The inset shows a detailed view of nerve bundles, and the ganglionic region collected for spatial proteomic analysis. Scale bar 250 μm. (C) Protein counts from isolated samples obtained using LCM-Proteomics. (n=4–6 cells/group, ** indicates p-value <0.01). (D) PCA plot of LFQ intensities reveal distinct clustering between nerve bundle samples and myenteric ganglions. (E) Overlap of identified proteins between samples and proteins uniquely identified in each group. (F) Distribution of differentially expressed proteins between nerve bundle samples and myenteric ganglions. (G) Bar graphs depict the mass spectrometry protein intensities of the synaptic proteins alpha-synuclein (aSyn) and vesicle-associated membrane protein 2 (Vamp2), both identified as differentially regulated between the two populations (as indicated in Fig. G). Statistical significance was determined using Student’s *t*-test (*p* < 0.05 is denoted by *). Immunofluorescent validation of aSyn and Vamp2 expression in the myenteric plexus highlights increased aSyn and Vamp2 puncta in ganglionic regions. Scale bar: 100 μm.

We extended our study to evaluate whether LCM-guided proteomics workflow could be effectively applied to the peripheral nervous system (PNS), focusing on neurons in the gut. These neurons are particularly relevant in neurodegenerative diseases like PD, where the gut-brain axis plays a critical role via PNS pathways(28). For this analysis, we isolated the longitudinal muscle with the myenteric plexus (LMMP) layer, which houses the myenteric plexus (MP) neurons (Fig. 4A). The MP network comprises axonal bundles and myenteric ganglia, the latter containing cell bodies interconnected by axons traversing the network (Fig. 4B).

Using this workflow, we attempted to isolate axonal bundles—structures typically challenging to study in the dense central nervous system (CNS)—and ganglia-containing cell bodies of comparable size. The protein counts were ~1500 for MP ganglia and close to ~1100 for the nerve bundle samples (using the DDA method) (Fig. 4C). We identified overall 1457 proteins across both populations, with an additional 431 proteins exclusively detected in ganglionic samples and 26 in axons neurons (Fig. 4D). Unsupervised PCA revealed two distinct clusters for these groups, highlighting significant proteomic differences between them (Fig. 4E). Interestingly, several synaptic proteins, were enriched in the MP ganglia (Fig. 4F). This observation aligns with the anatomical organization of the MP network, where axons encircle cell bodies within ganglia, creating synapse-rich regions. We validated the distribution of two synaptic proteins associated with the proper synaptic function(29) and neurodegeneration(30), alpha-synuclein (aSyn), and vesicle-associated membrane protein 2 (Vamp2) using IHC. Images show a greater density of bright punctate protein distribution at the ganglion compared to axon bundles (n=3 animals) (Fig. 4G). This finding is further supported by previous report(31). Additionally, many cytoskeletal (i.e., Cald1, Mapt, Mbp, Tpm1) and mitochondrial (i.e., Ndufa5, Atp5f1d, Uqcrb, Cyc1) proteins were abundant, possibly reflecting their general prevalence in neuronal structures and the surrounding muscle layer of the LMMP.

Collectively, these findings demonstrate that histology-guided microtissue proteomics can capture the biological differences between neuronal processes and ganglia in the PNS. This method holds promise for studying peripheral neuronal structures and processes that are challenging to dissect with traditional tissue-level omics approaches.

## Discussion

Spatial proteomics, particularly visual proteomics, which combines the ability to preserve spatial context while probing the proteome’s deep complexity, has recently emerged as a pivotal technology for understanding biological processes and their progression(32, 33). A key strength of this approach lies in its integration with well-established imaging techniques, including protein-based IF and RNA-based HCR (Fig 1B, Sup. Fig S1A–B), which have served as foundations for other spatial omics technologies. By leveraging these imaging strengths, this method can be readily adapted to target other cell types and tissues, therefore facilitating the investigation of cellular heterogeneity and the identification of cell-specific markers and pathways.

In this study, we highlight the capabilities of LCM-ScP as a versatile and robust tool in spatial proteomics, combining the power of advanced mass spectrometry with pathology-guided spatial resolution. Recent studies have used LCM-guided visual proteomics(34) to investigate organs that are much more homogenous and less complex than the brain. In this work, we use LCM-ScP in multiple core neuroscience problems to demonstrate how this method enables an unbiased single-cell proteomic analysis, a platform for discovery-driven investigations into previously uncharacterized nervous system biology.

Our primary analysis of neuronal subpopulations and neuro-glia comparisons demonstrated the ability of this method to effectively cluster brain region-specific neurons (Fig. 2E) and identify numerous neuron-specific markers with high confidence (Fig. 2, Sup. Fig. S4). Moreover, the study of neuroimmune responses in the stab wound injury (SWI) model provided critical insights. SWI is a commonly used TBI model to investigate neuroimmune responses in the brain. Bulk proteomic studies have identified protein changes linked to extracellular matrix (ECM) components(26). However, such proteins are underrepresented in our single-cell study, likely due to their extracellular localization. A recent study employed single-cell spatial transcriptomics to study SWI(27), enabling us to do a direct comparison of their significantly perturbed hits with our study. We identified a significant overlap between our protein hits and the transcriptomic hits identified by Koupourtidou et al.(27), with similar fold changes for almost all common proteins (i.e., Anxa3, B2m, Dhrs1, s100a6, Npl, Ptpn6, Isg15, C1qa, C1qc). In the same comparison, we observed CD68, a key protein marker of inflammation, showing greater enrichment at the protein level (~6 fold) in our study than at the transcriptomic level (~2.5 fold) reported by Koupourtidou et al. This reflects the localized nature of protein accumulation near the injury core (Fig S4C(i)), from where we collected our samples, while mRNA distribution is more diffused(27). Moreover, in the same work, staining for other mRNA and proteins imaging for selective proteins highlighted substantial differences in spatial distribution between mRNA and protein, strongly reaffirming that transcriptomics and proteomics are complementary rather than interchangeable(16, 35). These findings also underscore the importance of spatial proteomics in capturing injury-specific protein dynamics that may be missed by transcriptomics alone.

The PNS plays a pivotal role in maintaining body function by relaying sensory and motor signals between the central nervous system (CNS) and the rest of the body. Unlike the CNS, its architecture often features two distinct components: ganglia-containing neuronal cell bodies (e.g., nodose or celiac ganglia) and long axonal projections/nerve bundles extending to target tissues(36). Bulk proteomic analysis of whole gut tissue(37) or isolated LMMP layer(38, 39) lacks specificity by capturing proteins from non-neuronal components, particularly muscle tissue. While LCM has been used for mRNA studies(40) ganglionic nuclei, it is underexplored for proteomics at this resolution. Our staining-guided approach enabled precise collection of specific PNS regions, such as myenteric ganglia and associated axonal bundles, improving both flexibility and specificity in protein identification.

This approach revealed previously uncharacterized differences between ganglia and axonal regions. For instance, ganglionic fractions were enriched in synaptic proteins, including Vamp2, Homer1, and aSyn(31), reflecting the dense synaptic activity and varicosities formed within the ganglia(41, 42). These findings align with the ganglia’s role as a hub of synaptic integration in the PNS, where neurons synapse and form local circuits. The broader implications extend to understanding ganglionic biology and other units of the PNS, such as autonomic and sensory ganglia, which are critical in systemic regulation and pathologies involving peripheral nerves(43).

By leveraging spatial proteomics, we aim to uncover the distinct proteomic landscapes and spatial organization of CNS and PNS structures, offering insights into their roles in health and disease. However, some limitations of the LCM-ScP approach should be considered. First, we observed instances of background proteome contamination. For example, (i) attempts to isolate ramified astrocytes, which include their irregular protrusions, occasionally resulted in the identification of microglial proteins, especially during immune responses (Fig. 3, Fig. S4), and (ii) smaller cell types, such as microglia, presented challenges, particularly along the Z-axis, where excluding neighboring cell types is inherently difficult. Additionally, in the complex cellular architecture of the nervous system, where neuronal cell bodies are interwoven with dense networks of neuronal processes in the CNS or adjacent muscle tissue in the PNS, contamination from these surrounding structures is often unavoidable, as observed in this study (Fig. 2E, Fig. 5F). Balancing these trade-offs is crucial for achieving reliable and accurate analyses. Second, sample variability remains a challenge. High coefficients of variation (CV) were observed due to the inherently low sample size, which is typical for single-cell studies. While small sample sizes suffice to detect protein differences with large effect sizes—as demonstrated by some of our validated targets—larger sample sizes may be necessary to discern subtle differences. This is particularly important when distinguishing biological or disease-specific differences from variability introduced by methodological heterogeneity. For scenarios where cell-specific heterogeneity arises due to biological factors or disease states, deciphering these nuanced differences can be especially challenging. Despite these limitations, advances in mass spectrometry sensitivity, reduced costs for processing single-cell samples, and methodological improvements in LCM offer significant promise. These ongoing developments will likely enhance the potential of LCM-ScP to uncover novel targets and pathways, ultimately advancing our understanding of CNS biology and associated disorders.

In summary, this work has established spatial single cell proteomics as a powerful tool to study nervous system, opening possibilities to examine many biological questions and disease states. Recent advances in mass spectrometry sensitivity further enhance LCM-ScP’s ability to detect low-abundance biomarkers(15, 44), highlighting its value in discovery and translational research(45).

## Materials and Methods

### Intracranial stereotaxic surgery:

This study adhered to all applicable ethical guidelines, with all mouse procedures approved by the Institutional Animal Care and Use Committee (IACUC) at the California Institute of Technology. Mice were group housed under controlled conditions, maintained on a 13/11-hour light/dark cycle at ambient temperatures ranging from 71 to 75°F, with humidity levels between 30% and 70%. Prior to surgery, animals were anesthetized using isoflurane, and the animal was secured properly on the stereotaxic frame (Kopf Instruments). For the Stab wound injury (SWI) experiment, a needle tip (26 gauge) insertion was performed at the above-mentioned coordinate and left in place for 5 minutes.

### Post-mortem tissue processing and IF staining:

For IF staining, animals were euthanized using a sodium pentobarbital overdose and subjected to transcardial perfusion with chilled PBS, followed by 4% (w/v) paraformaldehyde (PFA) in PBS. The brains were then fixed for 24–48 hr. in PFA solution and subsequently cryoprotected at 4°C in a solution containing 30% (w/v) sucrose for 72 hr. Brains were flash-frozen in O.C.T. Compound (Scigen, cat#4586) using a dry ice-ethanol bath and kept at −70 °C until sectioning. Brian sections were obtained using a cryostat (Leica Biosystems) and collected in 1x PBS. Sections were stored at 4°C in PBS (supplemented with 0.02% Azide) for short-time storage or at – 20°C in cryoprotectant (Bioenno Lifesciences, cat 006799-1L) for longer preservation. Gut sections were stored in PBS supplemented with 0.02% sodium azide. The myenteric plexus neuronal layer was dissected out under a microscope. For IF staining, 35 μm free-floating brain sections were blocked with 10% (v/v) normal donkey serum in PBS supplemented with 1% Triton-X100 (% v/v) (1% PBST) for 90 min and then incubated with primary antibody solution prepared in 0.3% PBST supplemented with 0.3% (v/v) normal donkey serum overnight at 4°C. After washing in PBS (3 × 10 min), the sections were incubated with secondary antibodies conjugated with Alexa fluorophores (Jackson ImmunoResearch Laboratories, 1:500 dilution) for 90 min at room temperature and then washed 3 × 10 min in PBS. The tissues were mounted on glass slides or metal frame slides with PET membranes (Leica Microsystems, cat#76463-322) for confocal imaging and LCM, respectively. Glass slides were allowed to dry overnight and sealed with a coverslip using mounting media (Thermo Fisher Scientific, cat #P36970). Following primary antibodies used for the IHC staining: Anti-tyrosine hydroxylase (EMD Millipore, cat# AB152, 1:1000; cat#MAB318, 1:1000); anti-GFAP (Abcam, cat#ab4674, 1:1000), anti-Iba1 (FUJIFILM Wako Pure Chemical Corporation, cat#019-19741, 1:1000), anti-CD11b (Thermo Fisher Scientific, cat#14-0112-82, 1:500), anti-CD68 (Thermo Fisher Scientific, cat#14-0688-82, 1:500), anti-NeuN (Cell Signaling Technology, Cat#94403, 1:1000; Abcam, cat#ab104224, 1:1000) cat#), anti-Anxa1(Thermo Fisher Scientific,, cat#71-3400, 1:250), anti-Aldh1a1 (Thermo Fisher Scientific, cat#PA5-17943, 1:250), anti-PGP 9.5 (Thermo Fisher Scientific, cat#PA1-10011), gamma-synuclein (Genetex, cat#GTX110483, 1:500), Camk2a (Abcam, cat#ab52476, 1:500), anti-aSyn (Thermo Fisher Scientific, cat#32-8100, 1:500), anti-Vamp2 (Proteintech, cat#10135-1-AP, 1:500). Images were collected on a Leica spinning disk confocal microscope.

### hybridization chain reaction (HCR) *on mouse tissue:*

Split initiator probes(46) against *Itpr1* were designed according to Jang *et al*.(47) and ordered from Integrated DNA Technologies. All washes and incubations were performed at room temperature and with gentle shaking unless otherwise stated. All wash and incubation buffers were prepared from RNase-free reagents. Free-floating 35 μm sections of mouse cerebellum were permeabilized in 1x PBS with 0.1% Triton-X100 for 1 hour. Sections were then incubated for 1 hr. at 37 °C in a probe hybridization buffer consisting of 2x SSC, 10 % ethylene carbonate (Sigma-Aldrich, cat#E26258), and 10% dextran sulfate (Sigma-Aldrich, cat#3730). Following equilibration in hybridization buffer, the samples were incubated for 16 hr at 37 °C in pre-warmed fresh hybridization buffer plus 2 nM of each probe. After probe hybridization, sections were washed twice for 30 min in stringent wash buffer (2x SSC, 30% ethylene carbonate) at 37 °C, then twice for 30 min in 5x SSC with 0.1 % Tween-20 (Sigma-Aldrich, P1379), and then incubated in HCR amplification buffer (2x SSC, 10% ethylene carbonate) for 1 hr. Alexa Fluor 647-conjugated hairpins (Molecular Technologies) were heated to 95 °C for 90 s, then cooled to room temperature for 30 min in the dark. Following equilibration in amplification buffer, the tissue was incubated in amplification buffer with 60 nM hairpins for 16 hr. After HCR amplification, the samples were washed twice for 30 min each in 5x SSC with 0.1 % Tween20, followed by two washes in 1x PBS for 10 min. Sections were stored in 1x PBS at 4 °C until use.

### Laser-capture micro-dissection (LCM):

Regions of interest were excised from mounted tissues using a gravity-driven collection system by a Leica LMD7000(Leica Laser Microdissection Software Version V8.4). Laser settings are included in Supplementary table S3. The cut tissue was collected in a 0.65 mL Low Binding Tube cap (Sorenson SafeSeal, cat#11300) containing 10 μl of lysis buffer consisting of 50 mM triethylammonium bicarbonate (TEAB) (Thermo Scientific, cat#90114) and 0.1% n-Dodecyl-beta-Maltoside (DDM) (Thermo Scientific, cat#89903). Upon collection, the tube was vortexed upside down for 30 seconds and centrifuged at 13,000 × g for 1 minute at 25°C. The sample was then stored at −80°C until further processing.

### Sample preparation for mass spectrometry:

Stored samples were thawed and vortexed for 30 seconds in an inverted position with an additional 5 μL of lysis buffer to ensure the best recovery of the tissue. The sample was then centrifuged at 13,000 × g for 1 minute at 25°C and transferred into a LoBind 384 well PCR plate (Eppendorf, cat#0030129547). The plate was heated for two hours at 70°in a QuantStudio^™^ Real-Time PCR with the heated lid set to 85°C for sample lysis and protein denaturation. Following lysis, the plate was centrifuged at 1000 × g for 1 minute and allowed to cool to room temperature. Proteolytic digestion was carried out using trypsin (Promega, cat#V5280) and Lys-C (Wako Chemicals, cat# 125–05061) mix, 1 μL of digestion mix containing 4 ng of each enzyme was added to each sample. The plate was then incubated at 37°C overnight. Following digestion, samples were centrifuged at 1000 × g for one minute, and digestion was quenched with 0.5 μL of aqueous buffer comprising 2% acetonitrile and 4% formic acid.

### LC-MS/MS analysis:

Peptides were separated on an Aurora Ultimate UHPLC Column (25cm by 75μm, 1.7μm C18, AUR3–25075C18, IonOpticks) with column temperature maintained at 50°C. 5 μL of each sample was loaded onto the column. To optimize system sensitivity, peptides were directly introduced onto the analytical column. The separation gradient was configured with a flow rate of 0.22 μL/min and an analytical duration of 60 min. The LC system (Vanquish Neo UHPLC, Thermo Scientific) was coupled to an Orbitrap Exploris 480 mass spectrometer (Thermo Scientific) with a Nanospray Flex ion source (Thermo Scientific).

Data-dependent acquisition (DDA) was carried out in positive ion mode using a positive ion voltage of 1600 V while maintaining the ion transfer tube at a temperature of 300°C. MS1 scans were acquired with a range of 375–1200 m/z and a resolution of 60000 with a cycle time of 3 seconds. The maximum injection time was set to auto, and the normalized AGC target was set to 300%. Precursor ions with charges ranging from +2 to +6 were selectively targeted for fragmentation using a minimum intensity threshold of 5e3. Dynamic exclusion was set to exclude after one acquisition, with a 45-second exclusion duration and 10 ppm mass tolerance. MS2 scans were acquired in the Orbitrap at 60000 resolutions with an isolation window of 1.6 m/z, HCD collision energy set at 28%, and an auto-adjusted maximum injection time. The normalized AGC target was set at 200%. Xcalibur software (Thermo Scientific) was used for method implementation and data acquisition.

Similarly, data-independent acquisition (DIA) was carried out in positive ion mode using a positive ion voltage of 1600 V while maintaining the ion transfer tube at a temperature of 300°C. DIA settings were modified from Matzinger *et al.*(21). Briefly, MS1 full scans were acquired with a range of 375–1200 m/z and a resolution of 120000. The maximum injection time was set to auto, and the normalized AGC target set to 300%. MS2 scans were acquired at 60000 resolutions with a maximum injection time of 118 ms and AGC target of 75%. The scan range and further information on the gradient conditions and MS settings can be found in the supplementary information (Table S1 and S2).

### Mass spectrometry data analysis and statistics:

For RAW files obtained in DDA mode, Proteome Discoverer 2.5 (Thermo Fisher Scientific) using the SequestHT(48) search algorithm with Percolator validation. Data was searched against the mouse reference database from Uniprot (SwissProt database, downloaded 27 September 2024, 17232 sequences). Lys-C and Trypsin were selected, allowing for a maximum of two missed cleavages and peptide lengths between seven and 30 amino acids. The mass tolerance for precursor ions was set at 20 ppm, while the fragment mass tolerance was defined as 0.1 Da. INFERYS Rescoring was used in automatic mode, and Percolator was used for search validation based on q-values with a strict false discovery rate (FDR) of 1% at the spectrum level(49, 50). Carbamidomethylation of cysteine residues was designated as a static modification, and oxidation of methionine residues was considered a dynamic modification. A minimum number of 1 peptide sequence (unique and razor) was required for protein identification. Proteins meeting a stringent FDR threshold of 0.01 were assigned as ‘high confidence’ and used for further analysis. Strict parsimony was used to group proteins. Data was exported from PD2.5 and used for further analysis. RAW files obtained in DIA mode were analyzed in Fragpipe (version 22.0) using the DIA_SpecLib_Quant workflow with default parameters. Default DIA-NN parameters were used in the analysis(51). Protein and peptide quantification tables were then exported and analyzed in python in the Visual Studio Code editor environment (version 1.73), with additional python packages: numpy, pandas, scipy, scviz, UMAP, scikit-learn, and UpsetPlot. Kolmogorov-Smirnov (KS) normality test was used to test distribution for whether proteins unique to certain conditions lay within the original sample distribution. Fold change was plotted using Prism 9. Statistical analysis, sample clustering based on principal component analysis and volcano plots were generated utilizing MetaboAnalyst 6.0.

## Supplementary Material

Supplement 1

1

## Data Availability

The data, code, protocols, and key lab materials used and generated in this study are listed in a Key Resource Table alongside their persistent identifiers (Supplementary table S4). The proteomics datasets are available upon request.
