# Supplementary material for: Molecularly-guided spatial proteomics captures single-cell identity and heterogeneity of the nervous system
Source: bioRxiv. 2025 Feb 10:2025.02.10.637505. Preprint. [Version 1] doi: 10.1101/2025.02.10.637505 (PMC11844393; doi:10.1101/2025.02.10.637505)
Supplement: 1 [file NIHPP2025.02.10.637505V1-supplement-1.pdf]

bioRxiv preprint doi: https://doi.org/10.1101/2025.02.10.637505; this version posted February 11, 2025. The copyright holder for this preprint (which was not certified by peer review) is the author/funder, who has granted bioRxiv a license to display the preprint in perpetuity. It is made available under aCC-BY 4.0 International license.

Supplementary figure S1

(A) **Fixed** vs frozen tissue

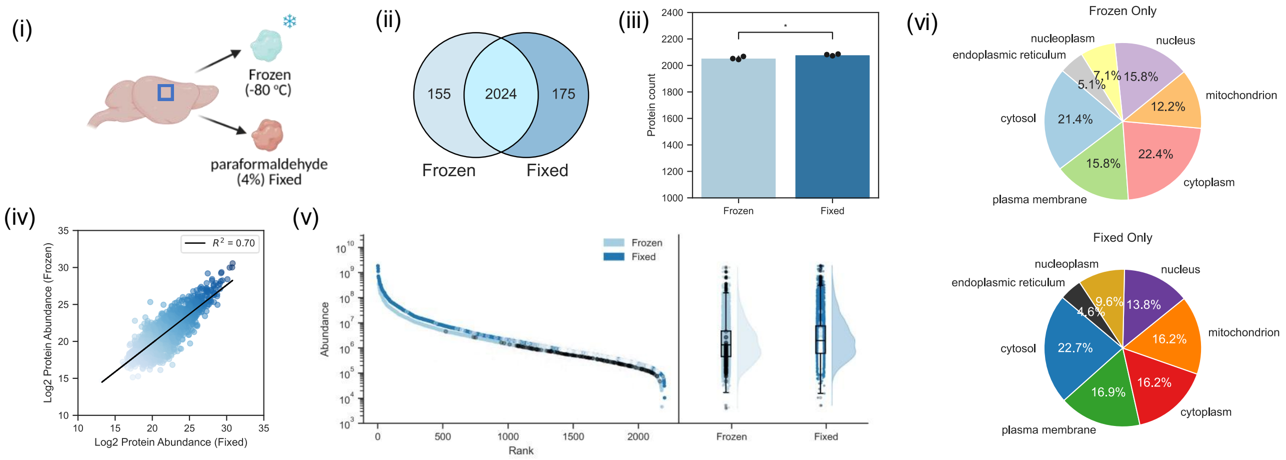

(B) **Protein detection** using IF staining

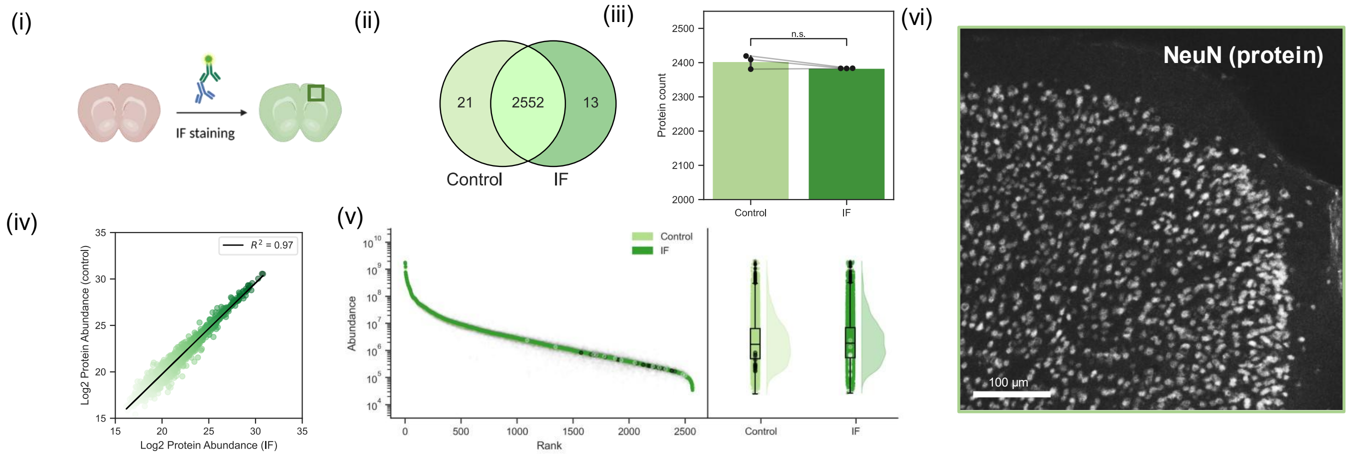

(C) **mRNA detection** using HCR

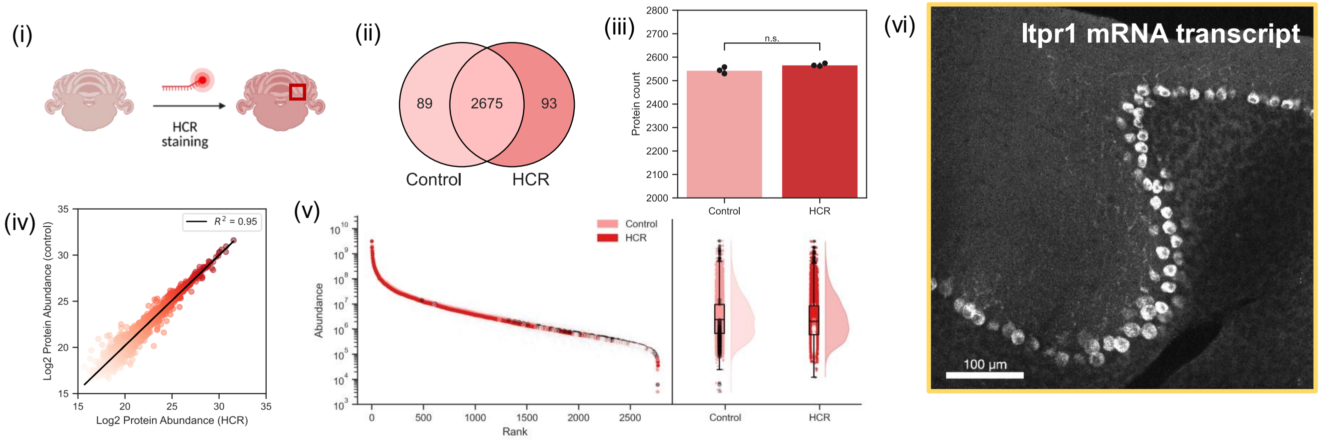

bioRxiv preprint doi: https://doi.org/10.1101/2025.02.10.637505; this version posted February 11, 2025. The copyright holder for this preprint (which was not certified by peer review) is the author/funder, who has granted bioRxiv a license to display the preprint in perpetuity. It is made available under aCC-BY 4.0 International license.

Supplementary figure S1: Assessment of proteomic coverage in staining-guided sample processing from fixed tissue.

(A) *Fixed versus frozen tissue:* (i) Schematic illustrating isolated frozen (-80°C) and4% PFA-fixed CNS tissue used in this comparison (ii) Overlap in protein identifications between frozen and fixed tissue. (iii) Protein counts, with statistical significance assessed by Student's t-test (p < 0.05 is denoted by *). (iv) Log2 protein abundance correlation between frozen and fixed tissue, with Pearson's R^2 values. (v) Rank-quant plot comparing protein abundance, with overlaid dots indicating uniquely identified proteins in frozen samples (black) and fixed samples (white). (vi) Pie chart showing the distribution of cellular components identified from gene ontology (GO) analysis of detected proteome in frozen and fixed tissue

(B) *Protein staining via IF:* (i) Schematic of IF staining. (ii) Overlap in protein identifications between IF and unstained (PBS) control condition. One hemisphere of a brain slice from three animals was immunostained and compared in a pairwise manner to the opposite unstained hemisphere from the same slice. (iii) Protein counts, with statistical significance assessed by Paired Student's t-test. (iv) Log2 protein abundance correlation between IF and control conditions, with R^2 values. (v) Rank-quant plot showing uniquely identified proteins, with overlaid dots indicating uniquely identified proteins in control samples (black) and IF samples (white). (vi) Image confirming Anti-NeuN immunostaining against NeuN protein.

(C) *mRNA staining using hybridization chain reaction (HCR):* (i) Schematic of HCR staining. (ii) Overlap in protein identifications between HCR and unstained (PBS) control conditions. (iii) Protein counts, with Student's t-test used for statistical analysis. (iv) Log2 protein abundance correlation between HCR and control conditions, with R^2 values. (v) Rank-quant plot with uniquely identified proteins highlighted, with overlaid dots indicating uniquely identified proteins in control samples (black) and HCR samples (white). (vi) HCR staining image against Itpr1 transcript,

bioRxiv preprint doi: https://doi.org/10.1101/2025.02.10.637505; this version posted February 11, 2025. The copyright holder for this preprint (which was not certified by peer review) is the author/funder, who has granted bioRxiv a license to display the preprint in perpetuity. It is made available under aCC-BY 4.0 International license.

Supplementary figure S2

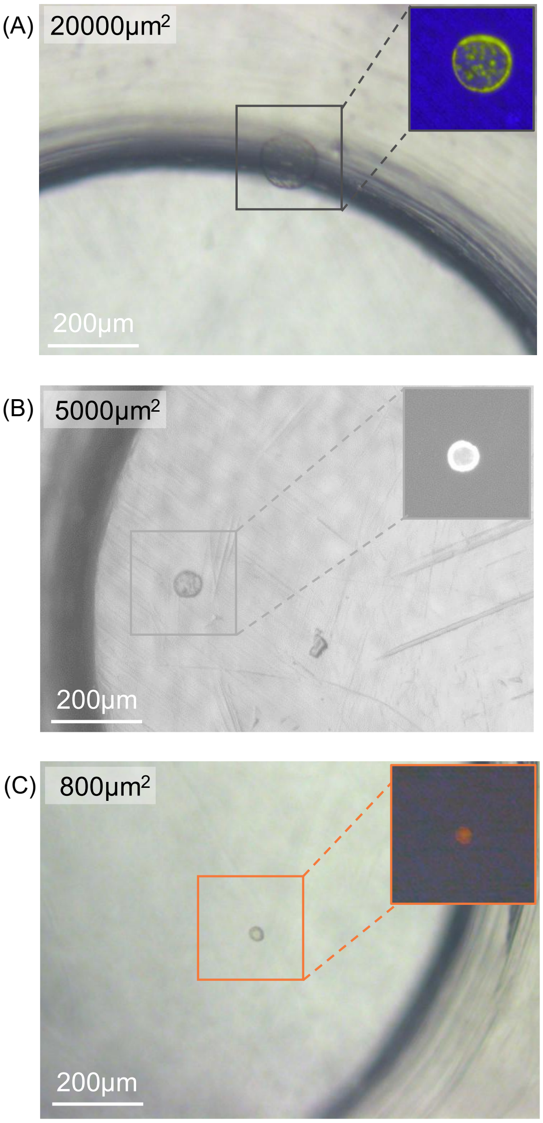

Supplementary figure S2. Visual confirmation of brain micro tissue samples after ROI collection. (A) Representative image of a 20,000 µm² tissue section collected in a tube cap (inset: fluorescent image, 488 channel) (B) Representative image of a 5000 µm² tissue section collected in a tube cap (inset: fluorescent image, Cy3 channel) (C) Representative image of an 800 µm² tissue section collected in a tube cap (inset: fluorescent image, Cy5 channel)

bioRxiv preprint doi: https://doi.org/10.1101/2025.02.10.637505; this version posted February 11, 2025. The copyright holder for this preprint (which was not certified by peer review) is the author/funder, who has granted bioRxiv a license to display the preprint in perpetuity. It is made available under aCC-BY 4.0 International license.

Supplementary figure S3

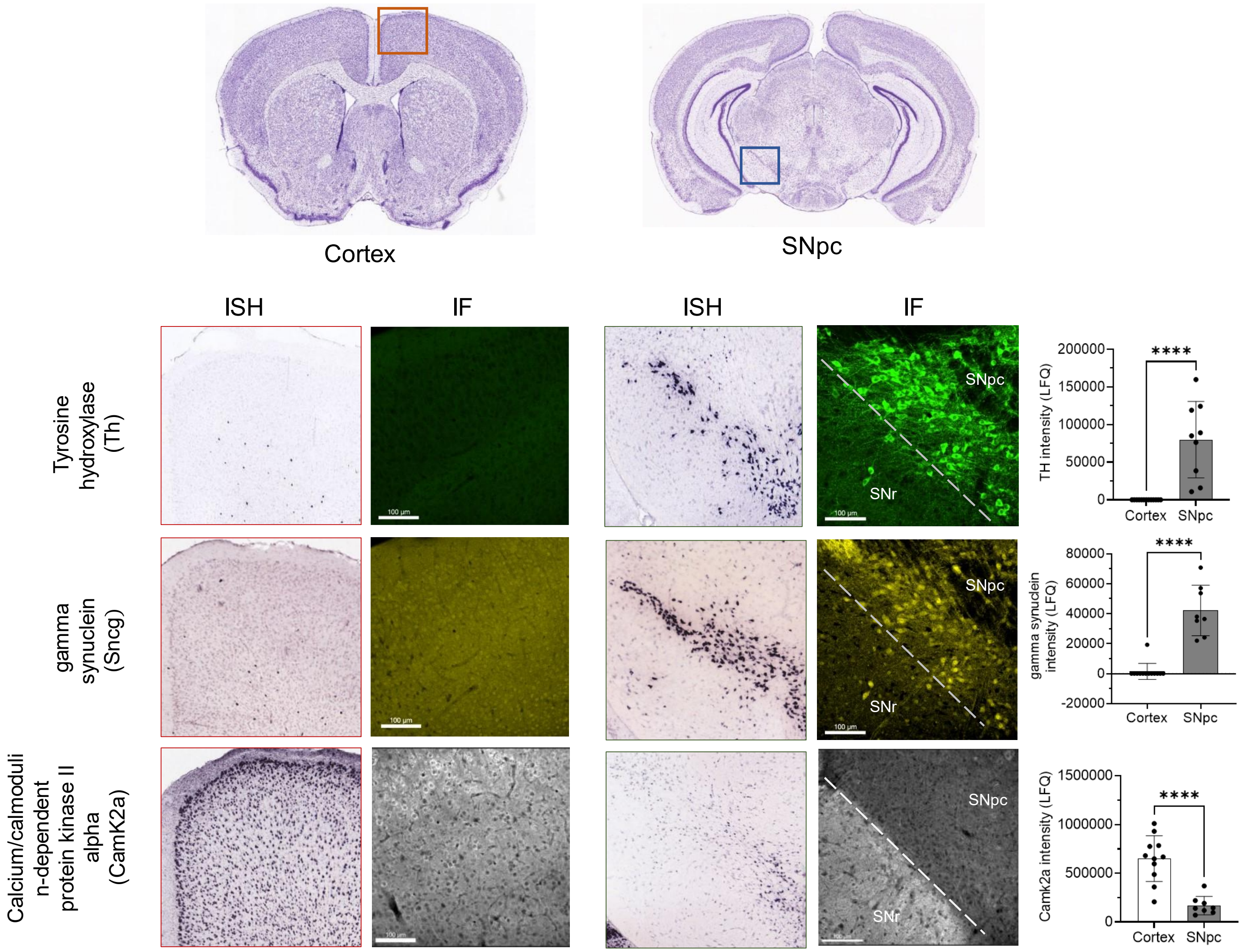

Supplementary figure S3: mRNA expression levels from the Allen Brain Atlas (ABA) and IHC validation of representative marker proteins identified in the SNpc and cortex: The red and green boxes on the Nissl-stained reference coronal brain section image indicate the approximate locations of the zoomed-in cortical and SNpc panels, respectively. Protein markers shown here are tyrosine hydroxylase (TH), gamma synuclein (Sncg), and calcium/calmodulin-dependent protein kinase II alpha (Camk2a).

Spatial in situ hybridization (ISH) data from the ABA illustrating the mRNA distribution are presented in the ISH columns (sourced from the mouse coronal ISH atlas published by the Allen Brain Initiative; TH- https://mouse.brain-map.org/experiment/show/1056; Sncg- https://mouse.brain-map.org/experiment/show/72081426; Camk2a-https://mouse.brain-map.org/experiment/show/79490122). Corresponding immunohistochemistry (IHC) validation using anti-TH, anti-gamma synuclein, and anti-Camk2a antibodies to indicate protein expression distribution is displayed in the IF columns. The respective bar graphs illustrate the mass spectrometry intensity levels of these marker proteins in each single-cell sample. Statistical significance was assessed using Student’s *t*-test ($p < 0.0001$ is denoted by ****).

bioRxiv preprint doi: https://doi.org/10.1101/2025.02.10.637505; this version posted February 11, 2025. The copyright holder for this preprint (which was not certified by peer review) is the author/funder, who has granted bioRxiv a license to display the preprint in perpetuity. It is made available under aCC-BY 4.0 International license.

Supplementary figure S4:

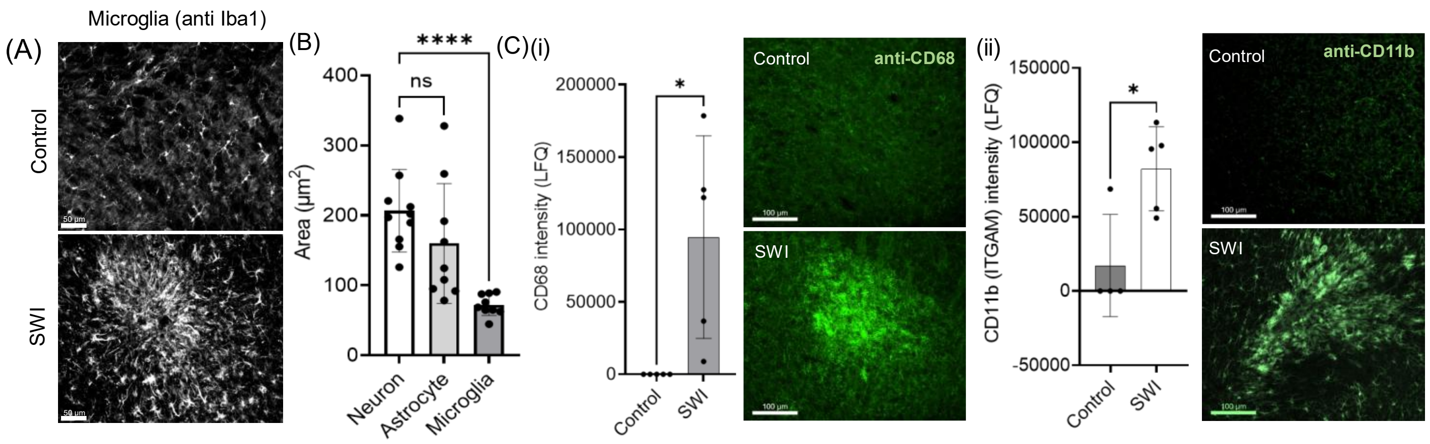

Supporting figure S4. Identification of reactive microglial markers at the site of acute injury

(A) Representative immunofluorescent images of microglia from control brain tissue and the site of acute injury. Iba staining highlights the presence of reactive microglia around the injury site.

(B) Quantification of single cell body areas isolable via LCM (measured from confocal images in Fig. 3A and S4A in control tissue, **** indicates p-value <0.0001).

(C) Bar graphs display the mass spectrometry-derived intensity levels of the identified reactive microglial marker proteins: (i) CD68 and (ii) Integrin Alpha M (CD11b). These proteins were observed to be significantly upregulated in samples from the injury site, as indicated in Figure 3G, compared to microglia-only samples. Immunohistochemical validation of CD68 and CD11b expression is shown for the site of injury. Statistical significance was assessed by Student's t-test (* indicates p-value <0.05).

bioRxiv preprint doi: https://doi.org/10.1101/2025.02.10.637505; this version posted February 11, 2025. The copyright holder for this preprint (which was not certified by peer review) is the author/funder, who has granted bioRxiv a license to display the preprint in perpetuity. It is made available under aCC-BY 4.0 International license.

Supplementary table S1: Gradient conditions for LC-MS/MS analysis

1 h gradient (51 min acquisition time)

Time (min)	Duration (min)	Flow rate (μl/min)	% Solvent A	% Solvent B
**0.0**	0.0	0.220	97.0	3.0
**1.0**	1.0	0.220	97.0	3.0
**31.0**	30.0	0.220	81.0	19.0
**41.0**	10.0	0.220	71.0	29.0
**51.0**	10.0	0.220	59.0	41.0
**52.0**	1.0	0.220	5.00	95.0
**52.0**	**Column Wash**			
**57.0**	5.0	0.220	5.0	95.0
**57.0**	**Stop Run**			
**57.0**	**Column Equilibration**			

bioRxiv preprint doi: https://doi.org/10.1101/2025.02.10.637505; this version posted February 11, 2025. The copyright holder for this preprint (which was not certified by peer review) is the author/funder, who has granted bioRxiv a license to display the preprint in perpetuity. It is made available under aCC-BY 4.0 International license.

Supplementary table S2: MS settings for DDA and DIA LC-MS/MS analysis

Settings	Full scan	DDA (ms2)	DIA (ms2)
**Resolution**	120000	30000	60000
**AGC target (%)**	300	200	75
**Maximum injection time (s)**	Auto	Auto	118ms
**Scan range**	375-1200		See below
**Isolation Window (m/z)**		1.6	See below
**RF Lens (%)**	50		
**HCD collision energy (%)**		28	30
**Intensity Threshold**	5.0e3		
**Charge States**	2-6		

Precursor mass range (m/z)	Isolation window (m/z)	Number of scan events
**375-500**	25	4
**500-600**	13	7
**600-800**	8	31
**850-900**	13	3
**900-1200**	25	9

bioRxiv preprint doi: https://doi.org/10.1101/2025.02.10.637505; this version posted February 11, 2025. The copyright holder for this preprint (which was not certified by peer review) is the author/funder, who has granted bioRxiv a license to display the preprint in perpetuity. It is made available under aCC-BY 4.0 International license.

Supplementary table S3: Leica LMD7000 Settings

Laser Settings

**Magnification**	20x	40x
**Power**	55	24
**Aperture**	3	1
**Speed**	3	12
**Specimen balance**	15	12
**Line spacing for Draw + Scan**	15	19
**Head current**	100%	100%
**Pulse frequency**	1510	1046
**Offset**	115	180
